# Book Review

**Published:** 1916-12

**Authors:** 


### BOOK REVIEWS

OUTLINES OF PHYSIOLOGY, by Edward Groves Jones, A.B., M.D., F.A.C.S., Professor of Surgery, Emory University, and Allen H. Bunce, A.B., M.D., Associate in Medicine, Emory University. Fourth edition, 373 pages, 111 Illustrations. Cloth, $1.50 net. P. Blaikiston's Son & Co., Philadelphia.

This small volume has come in a fourth edition in so short a time that its popularity is without question. It has earned this enviable state because of the skill with which its function as a compendium has been presented. The book makes no pretense to do more than recall in the briefest way consistent with accuracy the facts of physiology. It is of value to the student doing review work. It will be of assistance to him in reading Physiology for the first time because it gives a connected concept of the whole matter when a more extended work would confuse him. It makes a good reference book for the desk of the practitioner who sometimes needs a reminder of those fundamentals an active life has obscured.

There are a few errors in the references to the cuts in the section on neurons but these are typographic and not at all confusing.

---

THE PHYSICIANS VISITING LIST for 1917 includes an entirely new dose list prepared in accordance with the New U. S. Pharmacopoeia.

This will prove an exceedingly useful feature as there are many changes, improvements in standards, new drugs and other material inserted. This list gives the dose in both apothecary and metric systems and the solubility and important incompatibilities when called for.

Several other new tables have been inserted such as Isolation periods in infectious diseases, table of mortality, etc.

There are various editions as uusal, extending from 25 patients in one volume to 100 in two volumes. The prices run from $1.25 to $2.50 in the leather binding.

Published by P. Blaikiston's Son & Co., Philadelphia.

---

TEXT BOOK OF PHYSIOLOGY, Including a Section of Physiologic Apparatus, by Albert P. Brubaker, A.M., M.D., Professor of Physiology and Medical Jurisprudence in the Jefferson Medical College; formerly Professor of Physiology in the Pennsylvania College of Dental Surgery; formerly Lecturer on Physiology and Hygiene in the Drexel Institute of Art, Science and Industry. Fifth Edition, 776 Pages, 359 illustration, Cloth, $3.00 net. P. Blaikiston's Son & Co., Philadelphia.

Here we have a well-written book. Its style and diction are such that the student reader can take in the ideas without struggling with the media of their expression and wondering how the writer could have learned so many things and hidden them so cryptically as some of us had to do when pedantry was fashionable. We have gone over the several chapters with pleasure and relearned many things, but that which attracts us is the method of treatment of the various subjects in which fluency and directness vie for supremacy.

The book is primarily for the student and reader who is in his library and who wants his ideas formulated without doing laboratory work, himself. Thus many experiments and much apparatus have been left aside from the text or merely mentioned while the results, which the reader desires have been set forth. There is a Section on Laboratory work if one likes it, but that part of the study of physiology is not intruding itself to the confusion of one's mind.

The scientific matter of the various chapters is up-to-date and reliable. The chapters on Secretions and the Special Senses are particularily clear and satisfactory, and the book is commendable throughout.

